# Correction of Diabetic Erectile Dysfunction with Adipose Derived Stem Cells Modified with the Vascular Endothelial Growth Factor Gene in a Rodent Diabetic Model

**DOI:** 10.1371/journal.pone.0072790

**Published:** 2013-08-30

**Authors:** Guihua Liu, Xiangzhou Sun, Jun Bian, Rongpei Wu, Xuan Guan, Bin Ouyang, Yanping Huang, Haipeng Xiao, Daosheng Luo, Anthony Atala, Yuanyuan Zhang, Chunhua Deng

**Affiliations:** 1 Department of Urology, the First Affiliated Hospital of Sun Yat-sen University, Guangzhou, People’s Republic of China; 2 Wake Forest Institute of Regenerative Medicine, Wake Forest University, Winston Salem, North Carolina, United States of America; 3 Department of Urology, The Third Affiliated Hospital of Southern Medical University, Guangzhou, People’s Republic of China; 4 Department of endocrinology, the First Affiliated Hospital of Sun Yat-sen University, Guangzhou, People’s Republic of China; 5 Department of Urology, Dongguan People’s Hospital, Dongguan, People’s Republic of China; Medical University Innsbruck, Austria

## Abstract

The aim of this study was to determine whether adipose derived stem cells (ADSCs) expressing vascular endothelial growth factor (VEGF) gene can improve endothelial function, recover the impaired VEGF signaling pathway and enhance smooth muscle contents in a rat diabetic erectile dysfunction (DED) model. DED rats were induced via intraperitoneal injection of streptozotocin (40 mg/kg), and then screened by apomorphine (100 µg/kg). Five groups were used (n = 12/group)–Group 1 (G1): intracavernous injection of lentivirus-VEGF; G2: ADSCs injection; G3: VEGF-expressing ADSCs injection; G4: Phosphate buffered saline injection; G1–G4 were DED rats; G5: normal rats. The mean arterial pressure (MAP) and intracavernosal pressure (ICP) were measured at days 7 and 28 after the injections. The components of the VEGF system, endothelial, smooth muscle, pericytes markers in cavernoursal tissue were assessed. On day 28 after injection, the group with intracavernosum injection of ADSCs expressing VEGF displayed more efficiently and significantly raised ICP and ICP/MAP (*p*<0.01) than those with ADSCs or lentivirus-VEGF injection. Western blot and immunofluorescent analysis demonstrated that improved erectile function by ADSCs-VEGF was associated with increased expression of endothelial markers (VEGF, VEGF R1, VEGF R2, eNOS, CD31 and vWF), smooth muscle markers (a-actin and smoothelin), and pericyte markers (CD146 and NG2). ADSCs expressing VEGF produced a therapeutic effect and restored erectile function in diabetic rats by enhancing VEGF-stimulated endothelial function and increasing the contents of smooth muscle and pericytes.

## Introduction

Erectile dysfunction (ED) is one of the main complications in diabetes mellitus (DM). Approximately 20%–75% of diabetic men suffer from ED, which often occurs 10–15 years earlier and is more severe than that of non-diabetic men [1]. The causes of erectile dysfunction in men with diabetes are complex and involve impairments in blood vessel, muscle, and nerve function [2]. Vascular endothelial dysfunction, when the endothelium loses its physiological properties and shifts toward a vasoconstrictor, pro-thrombotic and pro-inflammatory state, is thought to play a major role in the early development of diabetic erectile dysfunction [3], [4]. Impairments in VEGF signaling system in the cavernosum appear to lead to diabetic erectile dysfunction (DED) [5], [6]. In addition, hyperglycemia may lead to smooth muscle dysfunction by the oxidation of low density lipoprotein and increased production of free-oxygen radical species [7].

Several treatment options are currently used for patients with DED. Oral medications (such as Tadalafil, Vardenafil, Sildenafil) are an important first-line treatment, but only 50–60% of these patients see improvements from these drugs [8] because the endothelial dysfunction caused by diabetes decreases production of nitric oxide (NO) [9]. Other therapies, including vacuum constriction devices, injection with alprostadil or papaverine hydrochloride, or implanting a penile prosthesis, have some advantages but also have limitations. Therefore, a novel therapy to restore impaired endothelial cells (ECs) and cavernous smooth muscle cell (SMCs) function is highly desirable for patients with DED.

Stem cell therapy is one of the promising strategies being investigated for patients with DED. Mesenchymal stem cells (MSC) are believed to be able to differentiate into various cell types including EC, SMC, Schwann cells, and neurons [10], [11]. These cells also are able to secrete paracrine factors and cytokines that enhance cell survival and angiogenesis, and promote anti-apoptotic, pro-neurogenic, anti-inflammatory, and anti-fibrotic effects [12], [13]. Intracavernous transplantation of bone marrow-derived stromal cells (BMSCs) had beneficial effects on erectile function of diabetic rats by increasing endothelium and smooth muscle content in the corpus cavernosum. Labeled BMSCs persisted in the penis until 4 weeks after injection [14].

Adipose-tissue derived stem cells (ADSCs) closely resemble BMSCs in differentiation and therapeutic potential, and are capable of expressing and secreting a broad spectrum of growth factors and cytokines [15]. Furthermore, the unique biology of autologous ADSCs including being easily expanded, immune privileged, and capable of long-term transgene expression even after multiple stages of differentiation, suggests their potential as a valuable gene delivery vehicle [16]. Autologous ADSCs injected into the penis of DED rats improved erectile function and decreased intracorporal tissue apoptosis, but very few labeled ADSCs could be found in the corporal cavernosum [17]. The therapeutic effect of ADSCs appear to be an indirect mechanism, whereby ADSCs improve the extracellular environment and local tissue function within the treatment area rather than through direct transformation of the ADSCs into local cell types [17]. Moreover, vascular endothelial growth factor (VEGF) is a cytokine with strong angiogenic properties. VEGF improved survival of transplanted MSCs in a myocardial infarction model [18]. A combination of stem cell therapy and gene therapy would more efficiently promote stem cell survival and increase cell differentiation efficiency for tissue repair [19].

The hypothesis of this study was that expression of VEGF by gene transfection could more efficiently improve the survival of ADSCs, restore endothelial function and increase smooth muscle content, which is able to recover erectile function in vivo. We delivered the VEGF_165_ gene into the ADSCs by lentivirus-VEGF_165_ [20] to correct diabetic erectile dysfunction and study the underlying mechanism in a rodent model.

## Materials and Methods

### Ethical Approval

A total of 77 ten-week old male Sprague-Dawley rats (260 g–320 g) were purchased from the Animal Center of Sun Yat-sen University and kept under standard laboratory conditions. The animal experimental protocol was approved by the Committee for Animal Care and Use of Sun Yat-sen University. Human fat tissue was harvested from orthopedic operations to assess the profiler of angiogenic proteins secreted by ADSCs because this ELISA kit (R&D Systems, Minneapolis, MN, USA) is designed to work in human tissues. The protocol to use human fat tissues and informed consent were approved by the Sun Yat-sen University Health Sciences Institutional Review Board. Written informed consent was obtained from the fat tissues donors.

### Isolation and Culture of ADSCs

Ten Sprague-Dawley rats were anaesthetized with pentobarbital sodium (30 mg/kg, ip) and adipose tissues were harvested from bilateral groin. Human or rat ADSCs were isolated according to our previously described protocol [20].

### Flow Cytometry Analysis and Multipotent Differentiation of Rat ADSCs

Cell surface antigens of ADSCs (passage 2) were determined by flow cytometry analysis. The cells were trypsinized and 5.0×10^5^ cells were washed and resuspended in flow cytometry buffer. Fluorescence-conjugated antibodies (**Table S1)** were incubated with ADSCs on ice for 30 min. Cells were then rinsed twice and detected by flow cytometry (Calibur BD Biosciences, Franklin Lakes, NJ), and the data were analyzed by using FlowJo vX software (Tree Star, Ashland, OR).

To determine the multipotent differentiation capacity of rat ADSCs, osteogenic and adipogenic differentiation were performed in induction medium for 3 weeks as following:

#### Osteogenic induction

ADSCs were seeded at 4,000 cells/cm^2^ and cultured in alpha MEM medium with 100 nM dexamethasone, 10 mM β-glycerophosphate, and 50 mM ascorbic acid-2-phosphate (Wako Chemicals, Richmond, VA) containing 10% FBS and 1% penicillin and streptomycin for 21 days. Differentiated osteocytes were then fixed by ice-cold 95% ethanol for 5 minutes at room temperature and stained for calcium deposits with 2% Alizarin Red Solution (PH 4.0). Light microscopy was used to identify orange-red stained areas, indicating calcium deposits.

#### Adipogenic induction

ADSC were seeded at 21,000 cells/cm^2^ and cultured in alpha MEM medium containing 10% FBS, 1% penicillin and streptomycin, 1 µM dexamethasone, 500 µM 3-isobutyl-1-methylxanthine, 10 µg/ml insulin, and 100 µM indomethacin for 21 days. Differentiated cells were then fixed with 4% paraformaldehyde for 30 min at room temperature and stained with fresh Oil Red O solution for 50 min. A light microscope was used to identify fat droplets.

### Angiogenic Trophic Factors Secreted by Primary Cultured Human ADSCs

A total of 2×10^5^ ADSCs at p2 were seeded in 6-well plates and then incubated with serum-free DMEM under normal conditions (5% CO_2_, 37°C) for 24 hours. The conditioned medium was collected and analyzed with a human angiogenesis array kit (R&D Systems, Minneapolis, MN, USA) according to the manufacturer’s instructions. Briefly, the membrane containing 55 angiogenesis-related antibodies was blocked with albumin for 1 hour on a rocking platform at room temperature. Membrane was then incubated with condition medium along with detection antibody cocktail overnight on a rocking platform at 4°C. The membrane was incubated with streptavidin-horseradish peroxidase conjugate antibody and developed on X-ray film following exposure to chemiluminescent reagents. Pixel density was measured by Quantity One® software (Bio-Rad. Life Sciences Research, Hercules, CA) and was normalized to the mean pixel density of the positive control spots on the same membrane.

### Transfection of Rat ADSCs by VEGF_165_ Lentivirus

Lentivirus expressing VEGF_165_-GFP or GFP alone was generated as previously described [20]. Multiplicity of infection (MOI) was determined as previously described [20]. To evaluate VEGF secreted by gene modified rat ADSCs, culture media of VEGF expressing ADSCs were replaced every 2 days and aspirated at days 1, 5, 10, 12, 15, 20, 25 and 30 for VEGF assays by ELISA according to the manufacturer’s instructions (R&D Systems).

### A Rat Model of Diabetic Erectile Dysfunction and *In vivo* Implantation

Type I diabetic erectile dysfunction was induced in rats by streptozotocin (STZ) according to our previously published methods [5]. Briefly, Sprague-Dawley rats received an intraperitoneal injection of STZ (40 mg/kg) after acclimatization for one week. Random plasma glucose levels were determined with an Accu-Check blood glucose meter (Roche, Mannheim, Germany) 2 days later. The rats with blood glucose levels higher than 300 mg/dl were regarded as diabetic. Eight weeks after STZ injection, Apomorphine (APO, 100 ug/kg) (Sigma) was used to screen the ED rats according to Heaton’s method [21]. After the APO subcutaneous injection, 48/55 (87.27%) rats were determined as DED rats.

For preparation of cell injection, about 4×10^6^ rat ADSCs (p3) were seeded in a 15-cm dish. About 4×10^7^ infectious units lenti-GFP/VEGF or lenti-GFP and polybrene (8 µg/ml) in 5 ml of DMEM (serum free) was added and incubated with ADSCs for 3 hours at 37°C, 5% CO_2_. The virus-containing medium was replaced with normal culture medium, and the ADSCs were cultured for another 48 hours before injection.

After the rats were anesthetized with pentobarbital sodium (30 mg/kg, ip), a total of 1×10^6^cells or lentivirus-VEGF (1×10^6^ infectious units) in 500 µl phosphate buffered saline (PBS) or 500 µl PBS only was injected into the corpus cavernosum using a 25 gauge needle. In addition, the blood drainage via the dorsal vein was halted at the base of the penis by circumferential compression with an elastic band before injection. The compression was kept and released one minute after the cell injection.

Five groups (n = 12 each) were used: Group 1 (G1) animals received an intracavernous injection of lentivirus-VEGF (1×10^6^ infectious units); G2 received an injection of GFP-labeled rat ADSCs (1×10^6^ cells); G3 received an injection of VEGF/GFP-expressing rat ADSCs (1×10^6^ cells); and G4 received an injection of 500 µl PBS as a negative control. G5 consisted of normal rats as a positive control. Totally five DED rats died and were replaced during the whole experiment.

### Erectile Function Assessment

Erectile function was determined by mean arterial pressure (MAP) and intracavernosal pressure (ICP) at days 7 and 28 as our previous report [5]. Briefly, rats were anesthetized with pentobarbital sodium (30 mg/kg, ip) and the left carotid artery was cannulated with a PE-50 catheter filled with 250 IU/ml heparinized saline to measure MAP. A heparinized (heparin 250 IU/ml) 25-G needle connected to another pressure transducer for physiological recording was inserted into one side of the penile crus. With a further midline abdominal incision, the cavernosal nerve was identified and isolated. A bipolar electrode attached to an electrical stimulator (ShangHai Biowill Co.,Ltd. Shanghai, China) was placed around the cavernosal nerve for applying stimulation. Monophasic rectangular pulses (the width was fixed at 0.2 ms, 1.5 mA, frequency at 20 Hz, and duration of 50 s) were delivered from the stimulator. Three electrostimulations were replicated at intervals of 10 minutes. Continuous simultaneous recordings of MAP and ICP were performed and recorded using a PC Lab (ShangHai Biowill Co.,Ltd. Shanghai, China) signal process system. Erectile responses were expressed as the ratio of ICP (mmHg)/MAP (mmHg)×100% [22]. Rats were sacrificed by using pentobarbital (200 mg/kg ip), after which penile tissue was immediately removed and divided into two parts. One part was fixed for paraffin sectioning or embedded in optimal cutting temperature (OCT), and the rest was frozen in liquid nitrogen for molecular studies.

### Histological and Immunohistological Analysis

The rat penile tissues were harvested and fixed in fresh 4% paraformaldehyde, dehydrated by an ethanol gradient, and then embedded in paraffin or in OCT. A series of 5 µm sections were immunohistologicaly analyzed with the primary (**Table S1**) and secondary antibodies. Quantitative image analysis was performed by computerized densitometry using the ImagePro program (version 6.3) coupled to a Leica microscope. For Masson Trichrome staining, 100× magnification images of the penis tissues composed of one half of the corpora cavernosa but excluding the sinusoidal spaces were analyzed for SMC (stained in red) and collagen (blue) and expressed as the ratio of SMC/collagen. For the quantitation of pericytes marker (CD146, NG2), ten fields of 400× magnification images were analyzed by two independent and blinded observers. The number of CD146, NG2 positive cells at X400 were counted and presented as the percentage of positive cells/total cells in the corpora cavernosa.

### Western Blotting

Penis protein samples were separated by 6%–12% sodium dodecyl sulphate polyacrylamide gel (15–30 µg/lane). Primary antibodies were applied after proteins were transferred to nitrocellulose membranes; β-actin was used as loading control. Signals were obtained in the linear range of detection and measured using a Fujifilm LAS-3000 imaging system (Minato-ku, Tokyo, Japan), and analyzed by Quantity One software® then presented as the relative density of each protein relative to beta-actin [23].

### Statistical Analyses

Values were expressed as mean ± standard deviation (SD). P values ≤0.05 were considered as statistically significant. Comparisons of weight, blood glucose, ICP, ICP/MAP, and Western blot analysis between the groups were performed by using one-way ANOVAs followed by a Student-Newman-Keuls post hoc test for multiple comparisons when appropriate.

## Results

### Characterization of Cultured ADSCs

The adherent cultured human and rat ADSCs presented a fibroblast-like morphology, maintained under non-stimulating conditions even after repeated subcultures to passage 20. These rat ADSCs were positive for CD73, CD90, CD105, and weakly positive for CD146. In addition, no expression of the hematopoietic and endothelial lineage markers (CD31, CD34, CD45, and CD117) was observed (**Fig. 1A**). The presence of adipogenic or osteogenic induced ADSCs (stained with Oil Red O or Alizarin Red Solution, respectively), further demonstrate the multipotential ability of primary cultured ADSCs (**Fig. 1B** **).**

**Figure 1:**
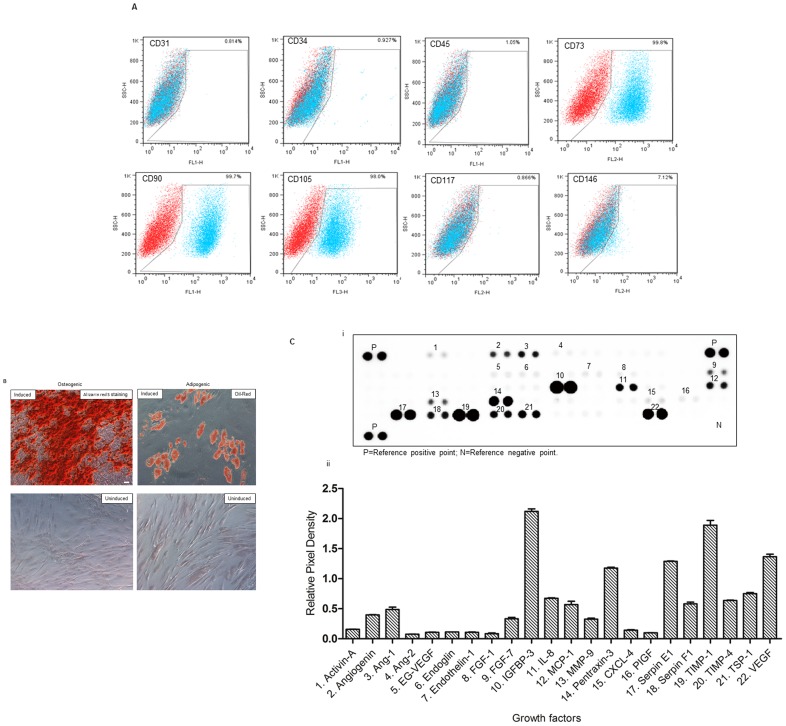
Characterization of rat adipose-derived stem cells (rADSCs). (A) FACS analysis of cultured rADSCs (*p*3) showed that surface antigens expression of rADSCs consistent with mesenchymal stem cell markers. Plots show the isotype control (red) versus specific antibody staining (blue). (B) Photomicrograph of osteogenic induced rADSCs (left; Alizarin red S staining) and adipogenic induced rADSCs (right; Oil Red O staining). Scale bar = 50 µm. (C) Proteomic profile angiogenesis array analysis of human ADSCs showed that 22 angiogenesis-related proteins were detected in the supernatant of human ADSCs.

### Angiogenic Factors Secreted by Human ADSCs *in vitro*

Twenty-two angiogenesis-related trophic factors including angiogenin, Ang-1, Ang-2, EG-VEGF, endoglin, endothelin-1, placental growth factor (PlGF), MMP-9, CXCL4, and VEGF were detected in the supernatant of human ADSCs (**Fig. 1C** **)**.

### Stable VEGF Expression of Rat ADSCs

Forty-eight hours after VEGF_165_/GFP was transduced into ADSCs by the lentivirus, its transduction efficiency reached >95% (percentage of cells displaying green fluorescence) (**Fig. 2A**). No significant difference in transduced ratio was found between VEGF_165_ and GFP transduced cells. The western blot analyses confirmed VEGF expression in VEGF165/GFP-transduced cells but not in GFP-transduced cells (**Fig. 2B**). In vitro VEGF secretion by VEGF expressing ADSCs in the culture medium peaked on day 12 post-infection and remained stable even on day 30 (**Fig. 2C**).

**Figure 2:**
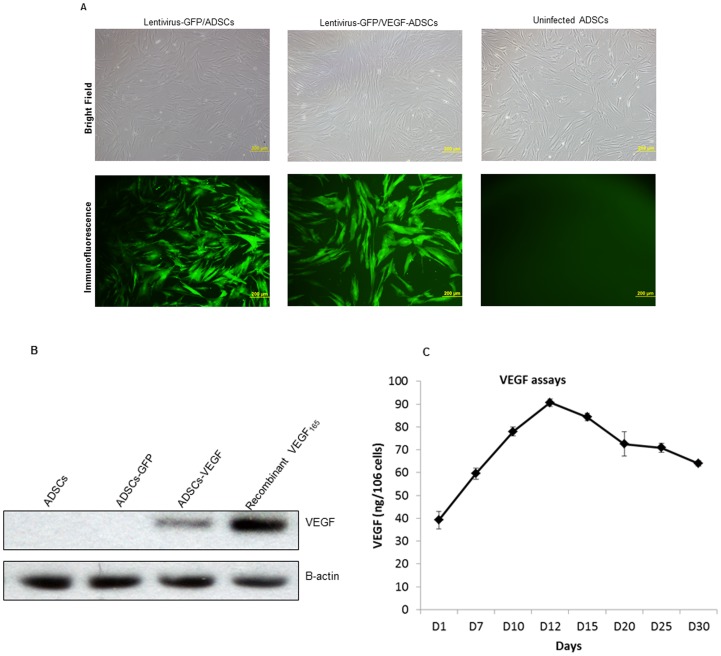
VEGF transfection to ADSCs delivered by lentivirus-VEGF. (A) Approximately 95% ADSCs expressed VEGF according to the immunofluorescent observation. Scale bar = 200 µm. (B) Western-Blot analyses further demonstrated the VEGF expression in ADSCs. (C) ELISA measurement showed VEGF protein levels in the culture medium of ADSCs reached peak at 12 days and kept stable level more than 30 days.

### Blood Glucose Concentration and Body Weight Changes

Compared with age-matched non-diabetic controls, STZ injection led to a significant increase of blood glucose levels (*p*<0.05) and caused body weight loss (*p*<0.05) in the diabetic-induced rats. After treatment, blood glucose concentration and body weights were not significantly different between untreated and treated groups (**Fig. 3**).

**Figure 3:**
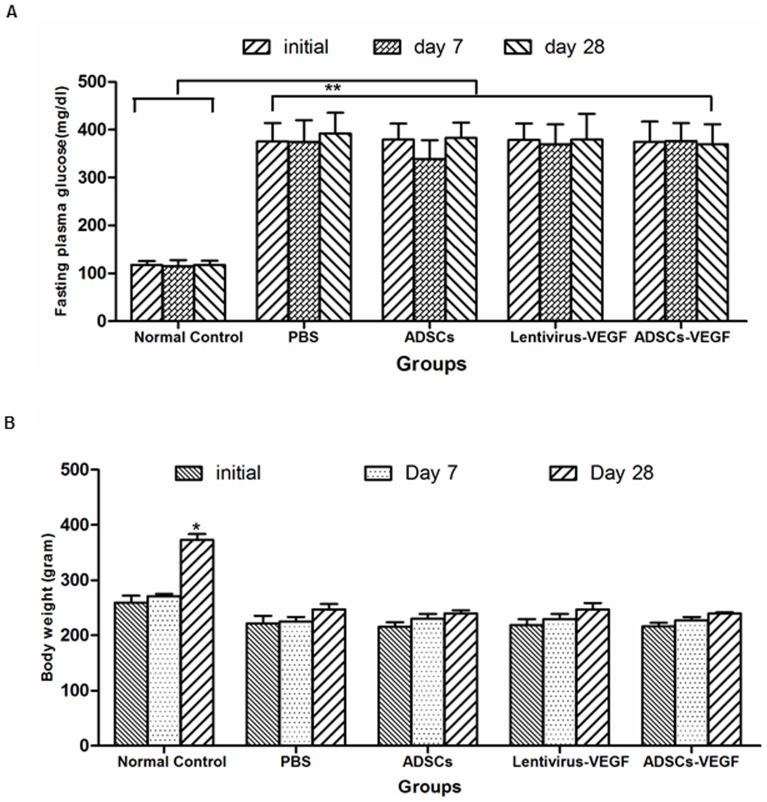
Physiologic and metabolic parameters after 4 weeks treatment. STZ injection led to a significant stable increase of random blood glucose level (A) and body weight loss (B) in the diabetic rats compared to age-matched non-diabetic controls. *p<0.05, body weight of normal rats control at day 28 *versus* other group rats; **p<0.01. Both of the blood glucose concentration and body weights in diabetic rats were not affected by either treatment including VEGF or ADSCs or ADSCs-VEGF injection into the cavernous of DED rats.

### Erectile Function Measurement

Intracavernosal pressure tracing response to the stimulation of the cavernous nerve was recorded in all groups (**Fig. 4A** **).** Eight weeks after induction of diabetes, a significant decrease in erectile function was detected in the untreated diabetic rats, displaying a significantly lower ICP and ICP/MAP than the age-matched non-diabetic controls (Figs. 3B, 3C) (*p*<0.05). ADSCs-VEGF exhibited protective effect on erectile parameters in the diabetic rats. ICP and ICP/MAP reached up to 83.68% of normal control values, but were still significantly lower than those non-diabetic controls (**Fig. 4B, C**). Correspondingly, the total ICP and ICP/MAP tended to be slightly increased in the VEGF or ADSCs treated diabetic rats compared with the saline-treated diabetic rats (*p*<0.05, **Fig. 4B, C**).

**Figure 4:**
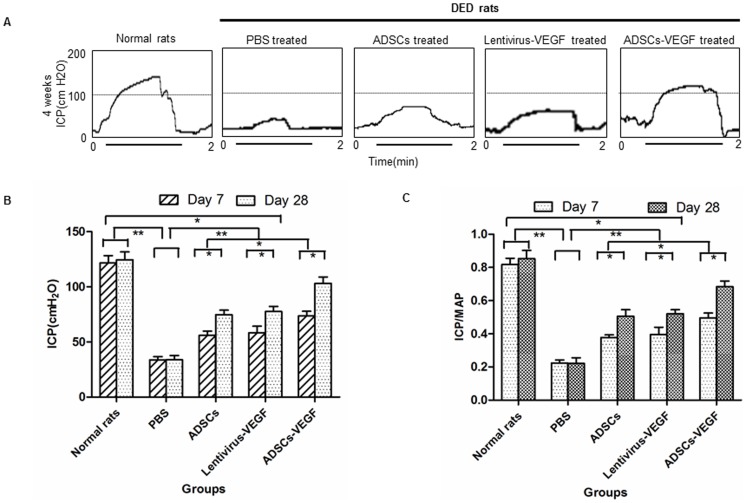
ADSCs-VEGF injection improved erectile function in streptozotocin (STZ)-induced diabetic rats. A: Representative intracavernous pressure (ICP) tracing response to the stimulation of the cavernous nerve (1.5 mA, 20 Hz, and duration of 50 s) in STZ-induced diabetic rats at 4 weeks after intracavernous injection of lentivirus-VEGF, ADSCs, ADSCs-VEGF; or untreated STZ-induced diabetic rats, and age-matched control rats. The stimulus interval (50 s) was indicated by a horizontal bar. B: The effects of treatment with ADSCs or VEGF or ADSCs-VEGF on the increase of ICP by the time. C: The ratio of total ICP to MAP was calculated for each group (n = 6). *p<0.05, **p<0.01. ICP = intracavernous pressure; MAP = mean arterial pressure.

### Expression of VEGF and its Receptor *in vivo*

Immunofluorescence staining and Western blot analysis showed that VEGF and its receptors VEGF receptor 1(VEGF R1), VEGF receptor 2(VEGF R2) in cavernous tissue were significantly less expressed in STZ-induced DED rats than in age-matched controls (*p*<0.05). Expression significantly increased in 4 weeks after injection of ADSCs-VEGF (G3) than in ADSCs (G2), lentivirus-VEGF treated DED rats (G1) and PBS-treated DED rats (G4) but was still lower than in age-matched controls (*p*<0.05). Moreover, ADSCs-treated (G2) and lentivirus-VEGF treated DED rats (G1) showed significantly higher increases in VEGF and its two receptors than PBS-treated DED rats. No difference in these markers was noted between ADSCs and lentivirus-VEGF treatments (**Fig. 5A**). Similar results were demonstrated by Western blot analysis (**Fig. 5B, C**). Furthermore, greater expression of VEGF, VEGFR1 and VEGFR2 was shown in G1, G2 and G3 on day 28 than on day 7 (**Fig. 5B, C**) (*p*<0.05). However, GFP-labeled transplanted ADSCs were not found in penis tissues of DED rats 28 days after cell implantation.

**Figure 5:**
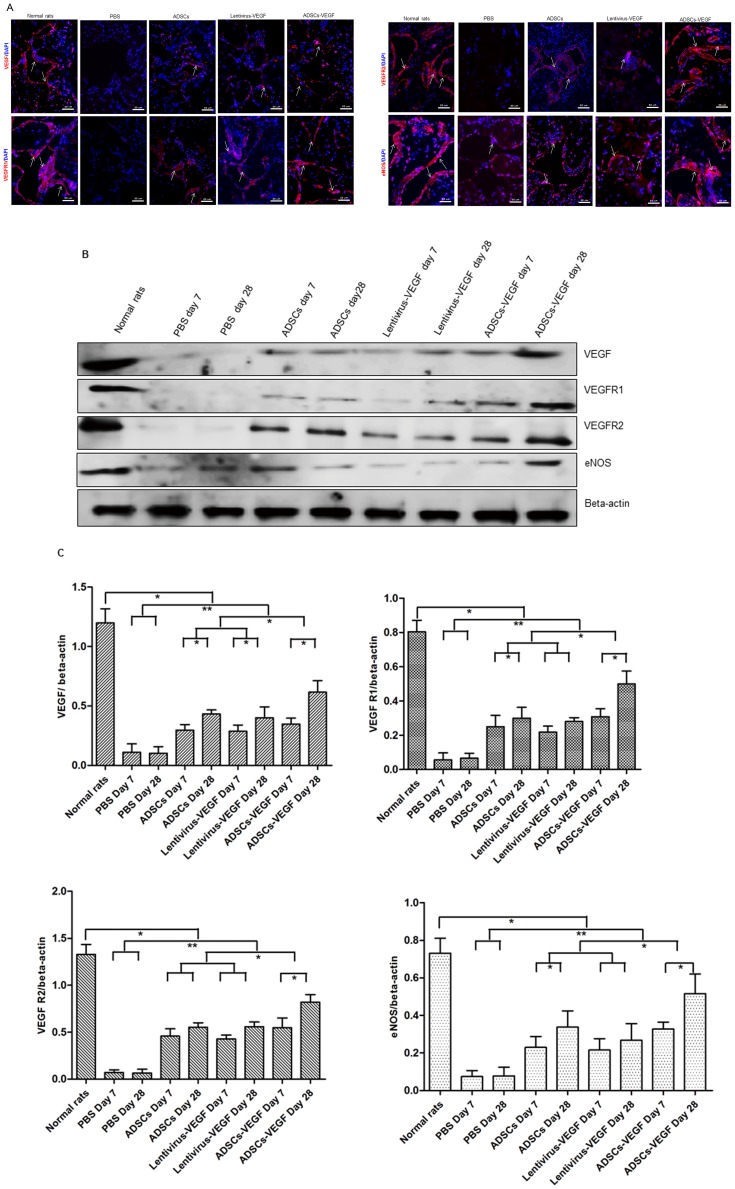
ADSCs -VEGF increased the expression of VEGF, its two receptors, and eNOS in STZ-induced diabetic rats. Expression of VEGF, its receptors VEGF R1 and VEGF R2, and eNOS increased in STZ-induced rats 4 weeks after ADSCs with or without VEGF or lentivirus-VEGF alone confirmed by immunofluorescence staining (white arrow represents specific staining) (**A**), Western blotting (**B**), and quantitative analysis for Western blotting (**C**). Expression was higher in the ADSCs-VEGF treatment group than with ADSCs or VEGF treatment, but no difference between ADSCs and lentivirus-VEGF treatment (**A–C**). VEGF, VEGFR1, VEGFR2 and eNOS expression were greater at 28 days after all three treatments than in 7 days (**B, C**). Scale bar = 50 µm; *p<0.05, **p<0.01.

### Expression of eNOS *in vivo*

There were significantly fewer cells expressing eNOS in cavernous tissue, as assessed by immunofluorescence staining, in the PBS-treated STZ-induced DED rats than in the age-matched controls (*p*<0.05, **Fig. 5A**). Decreased eNOS expression was not seen in groups with intracavernous administration of ADSCs-VEGF/GFP (G3), ADSCs (G2) or lentivirus-VEGF (G1) (**Fig. 5A**). In contrast, the ADSCs-VEGF treatment (G3) showed higher efficiency in the recovering eNOS than the other two treatments (G1 and G2). Similar to the results of immunofluorescence staining, immunoblot analysis of the corpus cavernosum tissues revealed a significant increase in cavernous eNOS expression in G1, G2, and G3 (*p*<0.05, **Fig. 5B, C**). Furthermore, eNOS protein levels increased with time (**Fig. 5B, C**), but no significant differences were found in eNOS levels between ADSCs (G2) and lentivirus-VEGF treatment (G1).

### Endothelial Marker Expression in Cavernous Tissues

Immunofluorescence staining and Western blot analysis showed that significantly fewer cells expressed endothelial markers (**Fig. 6A, B, C**) (CD31, vWF) in the PBS-treated STZ-induced DED rats than in the normal rats. On the contrary, endothelial content in the cavernous tissue after ADSCs-VEGF injection (G3) was almost completely restored. Furthermore, ADSCs or lentivirus-VEGF injection partially recovers in endothelial contents. Moreover, endothelial content was significantly increased on Western blot analysis in G1, G2, and G3 at day 28 compared with day 7 (**Fig. 6B, C**) (*p*<0.05).

**Figure 6:**
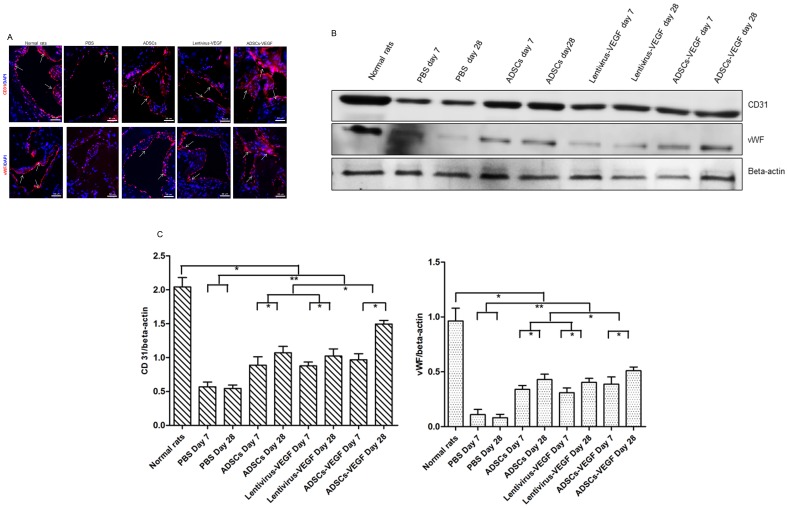
ADSCs-VEGF restored cavernous endothelial content in STZ-induced diabetic rats. A: Immunofluorescent staining of cavernous tissue using CD31 and vWF antibodies (showed by white arrow) in PBS-treated STZ-induced diabetic rats or ADSCs or lentivirus-VEGF or ADSCs-VEGF treated DED rats and normal rats. Scale bar = 50 µm. B: Immunoblotting analysis of the CD31 and vWF in the normal rats and STZ-induced diabetic rats after 1 weeks or 4 weeks treatment of PBS or ADSCs or lentivirus-VEGF or ADSCs-VEGF. C: Quantitative analysis of the immunoblotting by using Quantity One® software. *p<0.05, **p<0.01.

### Smooth Muscle Marker and Expression in Cavernous Tissue

The SMC-to-collagen ratio was reduced from 14.20±1.16% in the age-matched controls (G5) to 5.16±0.96% in the PBS-treatment (G4) group (*p*<0.01). After intracavernous administration of ADSCs or lentivirus-VEGF, the ratios were 8.14±0.99% and 7.58±1.27%, respectively. After ADSCs-VEGF injection, the ratio was 10.08±1.18% (*p*<0.05), indicating smooth muscle content was recovered (**Fig. 7A, B**). Expression of smooth muscles marker (α-smooth actin, smoothelin) in the cavernous tissue was significantly lower in the PBS-treated STZ induced DED rats (G4) than in the age-matched controls (G5). The smooth muscle markers in all treatment groups were still significantly lower than in normal control rats (*p*<0.05). The percentage of the smooth muscle markers was significantly increased after ADSCs-VEGF injection (G3) (**Fig. 7C**) but was slightly higher after ADSCs or VEGF treatment compared to controls (*p*<0.05). Western blot analysis also showed higher α-smooth actin and smoothelin protein levels on day 28 than on day 7 **(** **Fig. 7D, E** **)**.

**Figure 7:**
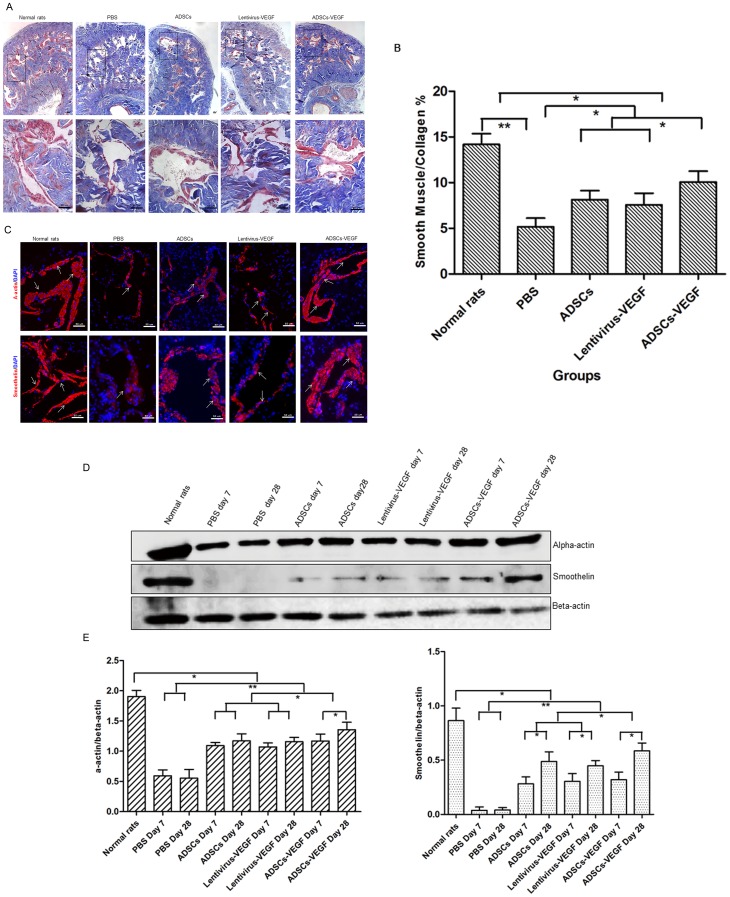
ADSCs-VEGF increased cavernous smooth muscle content in STZ-induced diabetic rats. Masson’s trichrome staining (A) and semi-quantitative evaluation (B) of smooth muscle/collagen ratio in the cavernous tissue showed this ratio was lower in PBS-treated diabetic rats than age-matched normal rats and was improved by ADSCs or lentivirus-VEGF or ADSCs-VEGF treatment. Scale bar = 50 µm; *p<0.05, **p<0.01. C: Immunofluorescent staining of cavernous tissue was performed with α-actin and smoothelin in age-matched control rats and STZ-induced DED rats treated with PBS or ADSCs or lentivirus-VEGF or ADSCs-VEGF. Scale bar = 50 µm. D: Representative Western blot for a-actin and smoothelin in normal rats and STZ-induced DED rats after 1 weeks and 4 weeks treatment with PBS, ADSCs, lentivirus-VEGF, or ADSCs-VEGF. E: Quantitative analysis of immunoblotting using Quantity One® software. *p<0.05, **p<0.01.

### Expression of Pericytes Markers in Cavernous Tissue

The numbers of the cells expressing pericytes markers (CD146 and NG2) in the cavernous tissue significantly decreased in DED rats (G4) compared with age-matched controls (G5), as confirmed by immunofluorescence staining with hemi-quantitative analysis (*p*<0.01, **Fig. 8A, B**). ADSCs (G2) or lentivirus-VEGF (G1) showed partial recovery of the numbers of pericytes in the DED rats. Obviously, the number of the cells expressing pericytes markers significantly increased after ADSCs-VEGF injection (G3) (*p*<0.05, **Fig. 8A,B**).

**Figure 8:**
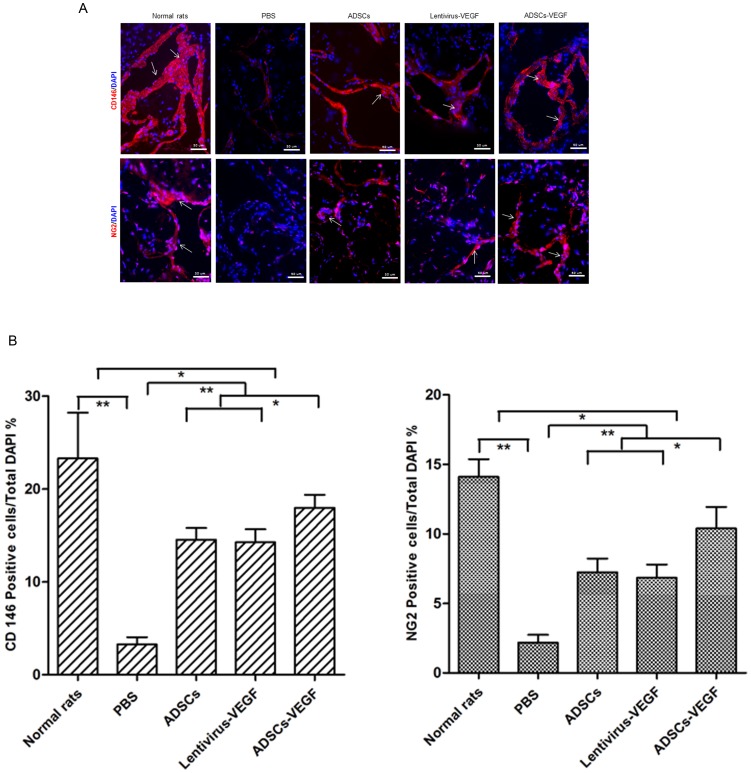
ADSCs-VEGF ameliorated pericytes markers in the cavernous tissue of DED rats. Immunofluorescent staining (A) and hemi-quantitative analysis (B) showed decreased pericytes markers (CD146, NG2) in the penis of STZ-induced DED rats treated by PBS compared with age-matched normal rats. And ADSCs or lentivirus-VEGF treatment partly but ADSCs-VEGF showed more effectively recovery of these markers. Scale bar = 50 um; *p<0.05, **p<0.01.

## Discussion

Erectile dysfunction is closely associated with impaired endothelial function and disruptions in the VEGF signaling pathway in a rat model of induced diabetes [5]. The phosphodiesterase type 5 inhibitors such as sildenafil can partially reverse the endothelial damages [5]. In this study, we found that cell therapy with ADSCs expressing VEGF accelerated the recovery of erectile function by increasing endothelial function and improving smooth muscle contents through its paracrine effects and VEGF expression, compared with ADSCs injection alone in diabetic rats.

Diabetic endothelial dysfunction is a contributing factor in the etiology of diabetic complications such as erectile dysfunction, retinopathy, nephropathy, and impaired wound healing [24]. The expression of VEGF and its receptors in diabetic pathogenesis is different among different organs or tissues, with higher expression in glomeruli, cerebral microvessels and retina of diabetic rats [25] but decreased expression in myocardial vessels in a murine model of diabetic cardiomyopathy [26] and in DED rats as well [6]. The mechanisms of endothelial dysfunction in response to sustained hyperglycemia include: 1) Increased oxidative stress and generation of superoxide radicals [27]: oxidative stress could activate several pathways including PKC, glycation of cellular DNA and other macromolecules, polyol, hexosamine and nuclear factor Kappa B pathways - all of which contribute to worsening of endothelial dysfunction in diabetes; 2) Defected VEGF signaling pathway [6]: diabetic endothelial dysfunction downgrades the VEGF signaling pathway. Restoration of this pathway supposedly plays an important role in the treatment of diabetic erectile dysfunction. In the present study, that the VEGF signaling system (including VEGF, VEGFR1, VEGFR2 and eNOS) worked poorly in diabetic penile tissues as a result of reduced expression of endothelial protein markers, leading to endothelial dysfunction-related erectile function damage, which is in agreement with Jesmin et al [6]. In the cavernous tissue of diabetic rats, expression of endothelial markers (including CD31 and vWF), accompanied by an impaired VEGF signaling pathway (including diminished VEGF, VEGFR1, and VEGFR2) were significantly reduced. Reduction of endothelial functional proteins (eNOS and endothelial markers) in the corpora cavernosa of diabetic rats may be explained by the presence advanced glycosylation end products, a heterogeneous group of nonenzymatically glycated proteins, lipids, and nucleic acids that may result in a loss of endothelial cells following oxidative stress [28], [29]. Erectile dysfunction could be more significantly recovered by enhancement of endothelial function via ADSCs-VEGF injection, which is more efficient than using ADSCs or lentivirus-VEGF.

Structural alterations in cavernous tissue, such as reduced smooth muscle content with an exaggerated deposition and disorganization of collagen deposition, are another important etiology for DED [30]. The presence of normal corporal smooth muscle content is a prerequisite for normal erectile response, and a reduction in corporal smooth muscle content leads to erectile dysfunction in diabetic rats [31]. In this study, the SMC-to-collagen ratio in cavernous tissue was reduced from approximately 14% in the normal rats to about 5% in the DED rats. After intracavernous administration of ADSCs or lentivirus-VEGF, it increased to almost 8% and with ADSCs-VEGF injection, to about 10%. The relaxation and nutrition of these smooth muscle cells may depend on the endothelial cells in corpora cavernosa of rat penis, and its deficit may be due to endothelial dysfunction in these diabetic rats.

A pericyte, a type of stem cells, is able to differentiate into smooth muscle cells and promote angiogenesis[32]–[34] and form vascular branches by direct communication between the cell membrane as well as paracrine signaling [35]. Interactions among endothelial cells, pericytes and vascular smooth muscle cells in the blood vessel acted as central processes in the regulation of vascular formation, stabilization, remodeling, and functioning. Failure of the interactions among these cells was seen in several pathological disorders, especially diabetic microangiopathy [36]. In addition, pericytes promote endothelial cell survival through secretion of diffusible angiogenic factors such as VEGF and Ang-1 [37]. In this study, both abnormal endothelial function and less cells expression of pericytes markers and smooth muscle markers were observed in diabetic cavernous tissues. Endothelial dysfunction in DED rats may also result from the impaired pericytes and its interaction with ECs. Moreover, the DED could be corrected by the ADSCs or ADSCs expressing VEGF, suggesting that recovery of both endothelial and smooth muscle function play an important role in enhancement of diabetic erectile dysfunction.

The multipotency of ADSCs has been utilized to repair muscle tissues and improve wound vascularization [15]. However, very few ADSCs could be successfully tracked after intracavernosum injection in a series of studies for ED treatments using stem cells, even though the function and structure of the animals were significantly improved [17], [38]. Those treatment effects may result from trophic factors such as CXCL5 secreted by implanted ADSCs [39]. In our study, we could not find GFP-labeled transplanted ADSCs in penis of DED rats after 28 days, even though the transfected VEGF gene should increase the survival of implanted cells [40]. A total of 22 angiogenesis-related cytokines, including angiogenin, Ang-1, Ang-2, EG-VEGF, endoglin, endothelin-1, VEGF, placental growth factor (PlGF), MMP-9, CXCL4 were detected in the culture medium of ADSCs. The angiogenin, Ang-1, Ang-2, EG-VEGF, Endoglin, Endothelin-1, VEGF and placental growth factor (PlGF) were the well-known pro-angiogenic factors [41], [42], which may help the recovery of endothelial function and erectile function of the DED rats in this study. Simultaneously, the erectile function demonstrated by ICP/MAP of DED rats could be recovered by unmodified ADSCs in comparison to the untreated DED rats, which is consistent with another study [17]. However, according to our data, rats with ADSCs transfected VEGF injection recovered erectile function and endothelial function better than those that received unmodified ADSCs or VEGF alone, indicating that ADSCs could also act as a novel gene delivery tools. Based on these data, ADSCs alone may be suitable for treatment of mild to moderate DED, while ADSCs expressing VEGF could be better for more severe cases.

Due to the safety concerns associated with lentivirus (including random insertional mutagenesis), in this study, we intended to demonstrate the syngeneic effects of ADSCs combined with VEGF. The VEGF gene manipulation used for proof-of concept in this study would not be used directly in the clinical applications. A safe pro-angiogenic delivery system has being investigated by this team for potential clinical application.

## Conclusion

In this study, we have demonstrated that impaired erectile function, abnormal VEGF signaling pathway, defect in endothelial function, smooth muscle content and pericytes were observed in STZ induced diabetic rats. Intracavernosum injection of ADSCs expressing VEGF has more efficiently promoted the recovery of the diabetic erectile function. Moreover, the potential mechanisms of these recoveries appear to be due to the rescued endothelial function produced by the paracrine effects of ADSCs and the external VEGF expression.

## Supporting Information

Table S1 Details of antibodies used in this study.
(DOC)
