# Supplementary material for: Correction of Diabetic Erectile Dysfunction with Adipose Derived Stem Cells Modified with the Vascular Endothelial Growth Factor Gene in a Rodent Diabetic Model
Source: PLoS One. 2013 Aug 30;8(8):e72790. doi: 10.1371/journal.pone.0072790 (PMC3758339; doi:10.1371/journal.pone.0072790)
Supplement: Table S1 — Details of antibodies used in this study. (DOC) [file pone.0072790.s001.doc]

**Supplement Table**

| Table S1. Details of antibodies used in this study | | | | |
| --- | --- | --- | --- | --- |
| Primary Antibody | Company/Catalog # | Antibody Dilution | | |
|  |  | IF | WB | FACS |
| VEGF | Abcam /ab1316 | 1:100 | 1:1000 |  |
| VEGF Receptor 1 | Abcam/ ab2350 | 1:50 | 1:500 |  |
| VEGF Receptor 2 | Abcam/ ab2349 | 1:50 | 1:500 |  |
| eNOS | Abcam/ ab5589 | 1:100 | 1:500 |  |
| CD31 | Santa Cruz/SC-1506 | 1:50 | 1:250 |  |
| vWF | Dako /A0086 | 1:200 | 1:500 |  |
| α-smooth muscle actin | Abcam/ab5964 | 1:200 | 1:1000 |  |
| Smoothelin | Abcam/ab8969 | 1:100 | 1:250 |  |
| CD146 | Abcam/ab75769 | 1:250 |  |  |
| NG2 | Abcam/ab101807 | 1:300 |  |  |
| CD31-FITC | abdserotec/MCA1334F |  |  | 1:10 |
| CD34-FITC | Santa Cruz / sc-7324 |  |  | 1:5 |
| CD45-FITC | abdserotec/ MCA43FA |  |  | 1:5 |
| CD73-PE | BD / [551123](http://www.bdbiosciences.com/ptProduct.jsp?prodId=62403&key=CD73&param=search&mterms=true&from=dTable) |  |  | 1:5 |
| CD90-FITC | abdserotec/ MCA47FA |  |  | 1:5 |
| CD105-KLH | Thermo / PA5-12511 |  |  | 1:10 |
| CD117-PE | Santa Cruz / sc-70455 |  |  | 1:10 |
| CD146-PE | Santa Cruz / sc-18837 |  |  | 1:10 |
